# Multiplex PCRs for the specific identification of marsupial and deer species from faecal samples as a basis for non-invasive epidemiological studies of parasites

**DOI:** 10.1186/s13071-020-04009-1

**Published:** 2020-03-18

**Authors:** Anson V. Koehler, Yan Zhang, Tao Wang, Shane R. Haydon, Robin B. Gasser

**Affiliations:** 1 Faculty of Veterinary and Agricultural Sciences, The University of Melbourne, Parkville, Victoria 3010 Australia; 2 Melbourne Water, Docklands, Victoria 3001 Australia

**Keywords:** Deer, Faecal samples, Host species-identification, Marsupial, Parasite, PCR

## Abstract

**Background:**

The specific identification of animals through the analysis of faecal DNA is important in many areas of scientific endeavour, particularly in the field of parasitology.

**Methods:**

Here, we designed and assessed two multiplex PCR assays using genetic markers in a mitochondrial cytochrome *b* (*cytb*) gene region for the unequivocal identification and discrimination of animal species based on the specific amplification of DNA from faecal samples collected from water catchment areas in Victoria, Australia. One of these assays differentiates three marsupial species (eastern grey kangaroo, swamp wallaby and common wombat) and the other distinguishes three deer species (fallow, red and sambar deer). We tested these two assays using a total of 669 faecal samples, collected as part of an ongoing programme to monitor parasites and microorganisms in these animals.

**Results:**

These two PCR assays are entirely specific for these animal species and achieve analytical sensitivities of 0.1–1.0 picogram (pg). We tested 669 faecal samples and found that some previous inferences of species based on faecal morphology were erroneous. We were able to molecularly authenticate all of the 669 samples.

**Conclusions:**

We have established PCR assays that accurately distinguish the faecal samples of some of the prominent large mammalian herbivores found within a water catchment system in the state of Victoria, Australia. The multiplex assays for marsupials and deer produce amplicons that are easily differentiable based on their size on an agarose gel, and can be readily sequenced for definitive species authentication. Although established for marsupials and deer, the methodology used here can be applied to other host-parasite study systems to ensure data integrity. 
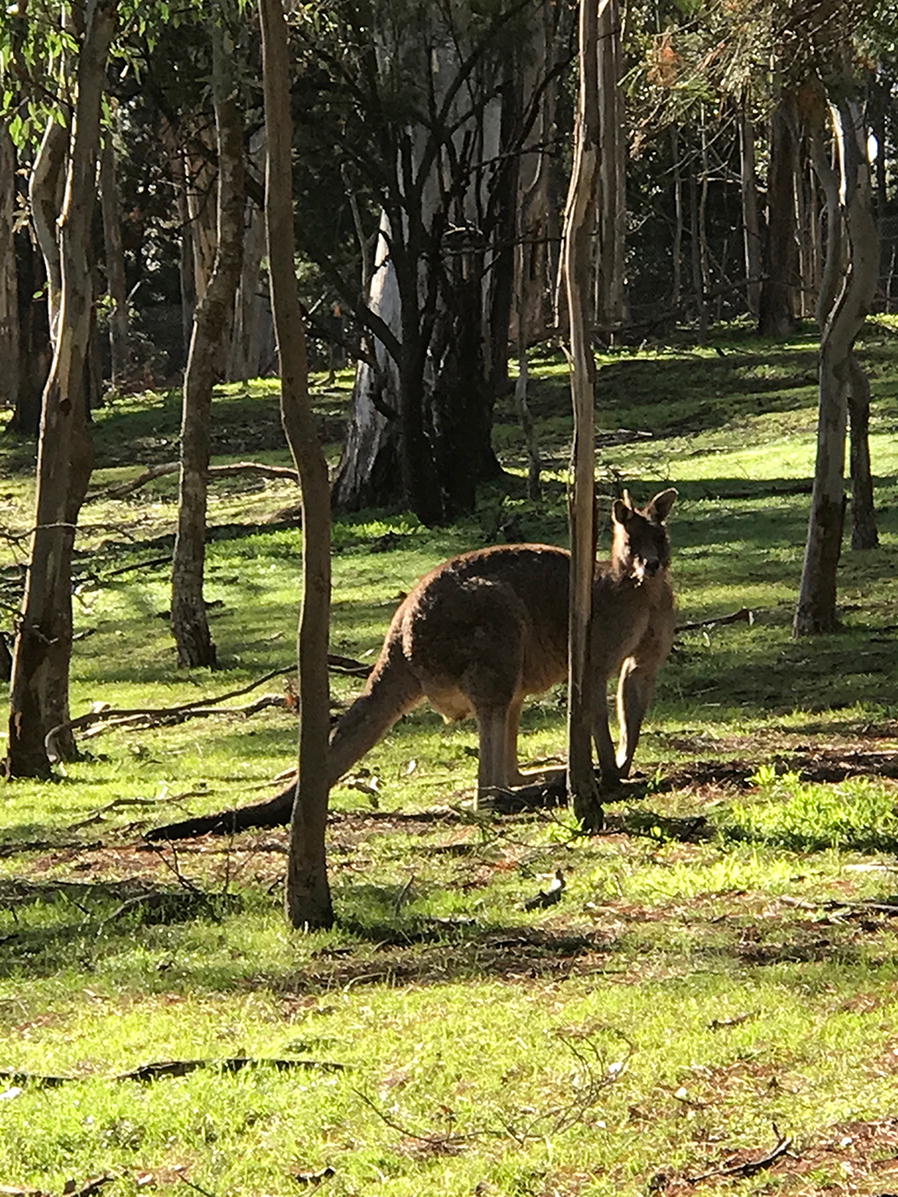

## Background

The non-invasive collection of wildlife faecal samples from the environment is a crucial research method to obtain precise data on parasites and other infectious diseases as well as host diet and genetics [1–6], and the accurate authentication of the host provenance of faeces is imperative for epidemiological and ecological investigations. Identifying the animal origin of faecal matter by morphological means is often challenging and can lead to misidentification and subsequent misinterpretation of associated data sets [6–8]. For example, the identification of species of ungulates based on faecal pellets is often ambiguous, because many factors, such as variation in diet, health status, size, age of an animal and/or season, can contribute to variation in faecal morphology [7–13]. Similarly, for large marsupials, marked variation in faecal morphology (e.g. clumping or unsegmented cylinders) relating to seasonal variability in diet is known to lead to challenges in the morphological differentiation of faeces among the eastern grey kangaroo (*Macropus giganteus*), swamp wallaby (*Wallabia bicolor*) and common wombat (*Vombatus ursinus*) [10] and between or among other marsupial species [14–16].

For epidemiological or ecological field studies, molecular techniques have been established for species identification from sources including faeces, feathers, hair, saliva, skin and urine [1–3]. Additionally, the biosecurity and food security industries have made great strides in developing techniques associated with species identification from unknown sources [17–20]. Such techniques include PCR-based restriction fragment length polymorphism (PCR-RFLP), multiplex PCR and quantitative PCR (qPCR)/real-time [6, 17–22]. For instance, a qPCR assay is reported to distinguish among the red fox (*Vulpes vulpes*), dog (*Canis lupus familiaris*) and cat (*Felis catus*), while simultaneously detecting *Toxocara* spp. and/or *Echinococcus multilocularis* DNA in faecal samples [6]. While conventional PCR-RFLP is known to lack diagnostic specificity [18–20, 23], multiplex end-point PCRs are consistently recognised as being specific, sensitive, time-efficient and cost-effective [2, 18, 20], and amplicons produced can be readily sequenced to confirm species identity. One strategy relies on the use of a common reverse primer in conjunction with unique (specific) forward primers, resulting in amplicons of varying sizes [2], which (if primers are designed and positioned well) are readily discernible on agarose gels [19]. We propose that this or a similar approach could be applicable to the identification and differentiation of faeces from species of wildlife in water catchments for the purpose of being able to match parasite species/genotypes with animal host species.

As part of an ongoing programme to monitor waterborne pathogens [24, 25], we have been routinely collecting faecal samples from wildlife (predominantly birds, canids, deer, marsupials and lagomorphs) from within 12 catchment areas located within the Yarra Ranges, Dandenong Ranges and Yan Yean and Greenvale catchment areas in the state of Victoria, Australia. Genomic DNA is extracted from individual faecal samples and eukaryotic microbes, including *Cryptosporidium* spp., *Enterocytozoon bieneusi* and *Giardia duodenalis*, are identified using molecular methods [25]. We have been inferring the host origins of faeces based on morphological criteria [10], but host species identification is sometimes unreliable. Key challenges relate to faecal samples from canids (fox and dog), deer (fallow deer (*Dama dama*), red deer (*Cervus elaphus*) and sambar deer (*Rusa unicolor*)) and marsupials (eastern grey kangaroo, swamp wallaby, common wombat) known to be prevalent in the water catchments.

To overcome this problem, we have developed multiplex PCRs to authenticate the origin of individual faecal samples from these animal species utilising genetic markers in mitochondrial (mt)DNA. We focused on using markers within the mitochondrial cytochrome *b* (*cytb*) gene, because (i) mt gene sequence (including *cytb*) data sets were publicly available for the six target animal species [26–30] and because the abundance of mtDNA in cells/samples would allow the development of assays with high analytical sensitivity.

## Methods

### Mitochondrial genomic data sets for six species of animals

Full mt genomes of each host species (marsupials: common wombat (NC_003322), eastern grey kangaroo (KY996502) and swamp wallaby (KY996500); deer: fallow deer (JN632629), red deer (AB245427) and sambar deer (MF177030)) were obtained from the GenBank database. Mt genome sequences were aligned in Geneious Prime 2019.1.1 (www.geneious.com) using MUSCLE alignment software [31].

### Samples from marsupials and deer

As part of an ongoing monitoring programme for waterborne pathogens [24, 25], we collected a total of 669 fresh faecal samples from marsupials and deer in water catchment areas of Victoria, Australia (Melbourne Water Corporation; between April 2019 and October 2019). We morphologically identified faeces as being from marsupials (*n* = 451) or deer (*n* = 188) using morphological criteria [10]. We also collected individual muscle samples from a common wombat, eastern grey kangaroo, swamp wallaby, fallow deer, red deer and sambar deer (‘target’ animals, identified by an expert zoologist, AVK), and goat and rabbit (controls) for the isolation of DNA samples for assessments of the specificity of PCR primers and assays; meat samples were collected from road killed animals (DELWP permit no. 10008033) or purchased from local supermarkets.

### Isolation of DNA from individual faecal or muscle samples

Genomic DNA was isolated from 0.25 g of each of the 669 faecal samples using the DNeasy Powersoil kit (Qiagen, Hilden, Germany) according to the manufacturer’s instructions. All 669 samples were tested by PCR (using the conditions described below) with either the marsupial or deer primer set. Control DNA was extracted from muscle samples from each common wombat, eastern grey kangaroo, swamp wallaby, fallow deer, red deer and sambar deer (‘target’ species), goat and rabbit using the same kit with the following alteration to the protocol: ~ 0.25 g of muscle was placed in 400 µl of extraction buffer (20 mM Tris-HCl (pH 8.0), 100 mM EDTA, and 1% SDS) and 20 µl of proteinase K (20 mg/ml; Promega, Fitchburg, Wisconsin, USA) and heated to 56 °C overnight. DNA amounts were estimated using a Qubit 3 Fluorometer (Thermo Fisher Scientific, Massachusetts, USA).

### Sequencing of the *cytb* gene from marsupials and deer species

From DNA of each of the three species of marsupial, the cytochrome *b* (*cytb*) gene (1146 bp) was PCR-amplified and sequenced using two pairs of overlapping primers. The first primer pair [forward (MarCBF 5′-TTT TAG YAT GGA CTC TAA CC-3′); reverse (MarCB3R 5′-GGT TGT TKG AGC CTG TTT CR-3′)] amplifies a 692 bp region, and the second primer pair [forward (MarCB5F 5′-TTY TCC GTR GAC AAA GCC AC-3′); reverse (MarCBR 5′-TGT TAA ATT ACT TGG ACT CTT CA-3′)] amplifies a 662 bp region. From DNA of each of the three species of deer, the *cytb* gene (1140 bp) was PCR-amplified and sequenced using two pairs of overlapping primers. The first set targets an 877-bp region using the primers 5DCytBF (forward: 5′-CGT TGT CAT TCA ACT ACA AGA ACA-3′) and 5DCytBR (reverse: 5′-TTG ATC GTA GGA TTG CGT ATG-3′). The second set targets a 784-bp region using the primers 3DCytBF (forward: 5′-TGA GGA CAA ATA TCA TTC TGA GGA-3′) and (DCytbR (reverse: 5′-TTT CTG GTT TAC AAG ACC AGT GT-3′).

PCR-amplification of *cytb* (in 50 μl reaction) was conducted using Go*Taq* buffer, 3.0 μM MgCl_2,_ 0.4 mM dNTPs, 50 pmol of each primer, 1.25 U of Go*Taq* polymerase (Promega, Madison, WI, USA) and DNA template - except for the negative (no-template) control. The cycling protocol was: 94 °C for 5 min (initial denaturation), followed by 35 cycles of 94 °C for 30 s (denaturation), 55 °C for 45 s (annealing) and 72 °C for 45 s (extension), with a final extension step at 72 °C for 5 min.

### Multiplex PCR for the delineation of marsupials (Mmars-PCR)

Mmars-PCR amplification was achieved in a 50 µl reaction (same reagents and concentrations as above) employing oligonucleotide primer pairs (Table 1) for eastern grey kangaroo (250 bp): forward (macF 5′-GCA TCC ATC TTA ATT CTC CTC A-3′) and reverse (marR 5′-GGT TYT AGT ATG TAG TTT TCA AA-3′); swamp wallaby (425 bp): forward (walF 5′-GCC CTA CTT TCA TTA GCA C-3′) and reverse (marR 5′-GGT TYT AGT ATG TAG TTT TCA AA-3′); and common wombat (289 bp): forward (VUCytBF 5′-AGC ATT CAT CGA CCT ACC CA-3′) and reverse (VUCytBR 5′-TGT TTC TTT GTA GAG GTA GGA G-3′) (Table 2) using the following cycling protocol: 94 °C for 5 min (initial denaturation), followed by 35 cycles of 94 °C for 30 s (denaturation), 59 °C for 30 s (annealing) and 72 °C for 30 s (extension), with a final extension step at 72 °C for 5 min. Known positive and negative (no-template) control samples were included in each PCR run, and aliquots of selected amplicons produced were routinely sequenced to confirm species-specificity of PCR amplification.

**Table 1:** Primers designed to target various portions of the mitochondrial cytochrome B gene (*cytb*) used to differentiate three marsupial and three deer species in two separate multiplex PCRs

Species	Primer	Sequence (5′-3′)	Product size (bp)
Eastern grey kangaroo	macF	GCATCCATCTTAATTCTCCTCA	250
	marR	GGTTYTAGTATGTAGTTTTCAAA	
Swamp wallaby	walF	GCCCTACTTTCATTAGCAC	425
	marR	GGTTYTAGTATGTAGTTTTCAAA	
Common wombat	VUCytBF	AGCATTCATCGACCTACCCA	289
	VUCytBR	TGTTTCTTTGTAGAGGTAGGAG	
Fallow deer	DDCytBF	AGCAACCTTAACTCGATTCTTC	197
	DDCytBR	AGAGAAATAGGAATAGGATGCC	
Red deer	CECytbF3	CGCAGACAAAATCCCCTTTCA	482
	CERUR	GTTTTCGATTGTGCTGGTGA	
Sambar deer	RUCytbF4	CCAGTGCCTATTCTGAATCTTAGC	161
	CERUR	GTTTTCGATTGTGCTGGTGA

**Table 2:** Summary of the BLASTn results for the sequenced amplicons of the mitochondrial cytochrome *b* gene (*cytb*) used to differentiate three marsupial and three deer species by testing faecal samples using two separate multiplex PCRs

PCR	Species	Product size (bp)	Match to sequence with GenBank ID	Sequence identity (%)
Mmars-PCR	Eastern grey kangaroo	250	EF368023	100
	Swamp wallaby	425	KJ868164	100
	Common wombat	289	MK360903	100
Mdeer-PCR	Fallow deer	197	MK575605	100
	Red deer	482	MF872247	100
	Sambar deer	161	MF177028	100

### Multiplex PCR for the delineation of deer (Mdeer-PCR)

Mdeer-PCR amplification was achieved in a 50 µl reaction (same reagents and concentrations as above) employing oligonucleotide primer pairs (Table 1) for sambar deer (161 bp): forward (RUCytbF4 5′-CCA GTG CCT ATT CTG AAT CTT AGC-3′) and reverse (CERUR 5′-GTT TTC GAT TGT GCT GGT GA-3′); fallow deer (197 bp): (DDCytBF 5′-AGC AAC CTT AAC TCG ATT CTT C-3′) and reverse (DDCytBR 5′-AGA GAA ATA GGA ATA GGA TGC C-3′); red deer (482 bp): (CECytbF3 5′-CGC AGA CAA AAT CCC CTT TCA-3′) and reverse (CERUR 5′-GTT TTC GAT TGT GCT GGT GA-3′) (Table 2) using a ‘touchdown’ approach: an initial denaturation at 94 °C for 5 min was followed by a denaturation at 94 °C for 30 s and an annealing step-down of 1 °C per cycle for 5 cycles, starting at 65 °C for 30 s, followed by an extension phase at 72 °C for 30 s. After the step-down cycling, there were 30 cycles of 94 °C for 30 s (denaturation), 60 °C for 30 s (annealing) and 72 °C for 30 s (extension), with a final extension step at 72 °C for 5 min. Known positive and negative (no-template) control samples were included in each PCR run, and aliquots of selected amplicons produced were routinely sequenced to confirm species-specificity of PCR amplification.

### Agarose gel electrophoresis and sequencing of PCR products

Aliquots (5 µl) of amplicons produced by multiplex PCR were resolved on ethidium bromide-stained 1.5% agarose gels using TBE (65 mM Tris-HCl, 27 mM boric acid, 1 mM EDTA, pH 9) as the buffer and using 100-bp DNA ladder (Promega, Madison, WI, USA) as a size marker. Amplicons were individually treated with thermosensitive alkaline phosphatase (FastAP) and exonuclease I (*Exo*I) (Thermo Fisher Scientific, Waltham, MA, USA), according to the manufacturer’s instructions, and subjected to Sanger sequencing (BigDye Terminator v.3.1 chemistry, Applied Biosystems, Foster City, CA, USA) using the same (forward or reverse) primers (individually) used for each species of animal in the PCR. The resultant sequences were compared with other sequences in the NCBI database using the option ‘BLASTn’.

## Results and discussion

### Sequence alignment, primer design and evaluation

First, we aligned complete mt genome sequences, and then identified gene regions with length and/or sequence variation among (but not within) animal species that were flanked by conserved regions for primer design. Our goal was to design forward and reverse primers (cf. [2]) that would produce specific PCR amplicons of < 500 bp that would differ in length by 40–50 bp among species, so that amplicons representing individual animal species were unique in size and differentiable from one another on an agarose gel (cf. [19]). After examining the fully aligned mt genomes, we observed that selected regions within the *cytb* gene met these criteria. Thus, we aligned all available complete *cytb* sequences for red deer (*n* = 250) with those accessible fallow deer (*n* = 8), sambar deer (*n* = 59), eastern grey kangaroo (*n* = 4), swamp wallaby (*n* = 4) and common wombat (*n* = 3), and designed primers (Table 1).

Secondly, we needed to verify that each of the primers for each animal species was consistent in sequence with its respective region in *cytb* determined from total genomic DNA from each of the six species of mammal from Victoria, Australia. Little (< 99.9 %) to no sequence variation in the primer regions was detected in *cytb* between sequences derived here from common wombat (accession no. MN746798), eastern grey kangaroo (MN746797) and swamp wallaby (MN746796) and those from GenBank (accession nos. NC_003322, KY996502 and KY996500, respectively). However, as marsupial population genetic studies have mainly utilised microsatellites and the control region [32–35], the number of *cytb* sequences available on GenBank is not extensive.

**Fig. 1:**
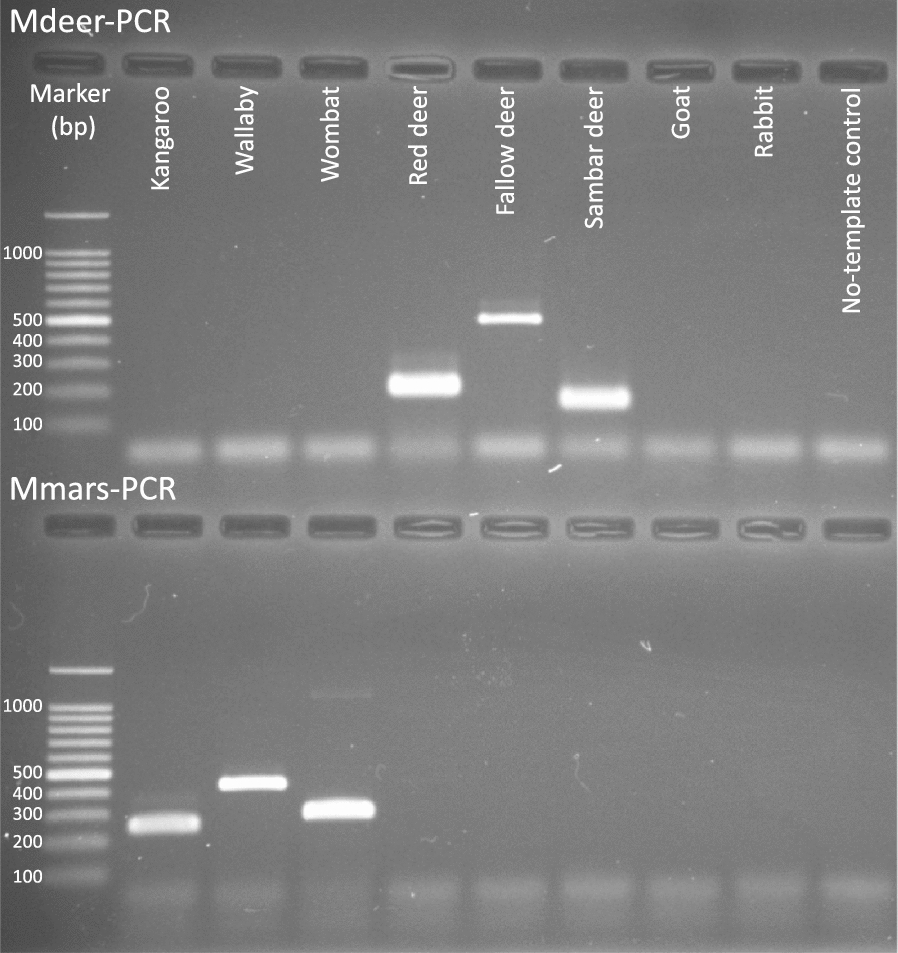
Mdeer-PCR and Mmars-PCR allow the unequivocal identification of cervid and marsupial species, respectively, present in Melbourne’s water catchment areas based on amplicon size: Eastern grey kangaroo (*Macropus giganteus*; 250 bp), swamp wallaby (*Wallabia bicolor*; 425 bp) and common wombat (*Vombatus ursinus*; 289 bp); fallow deer (*Dama dama*; 197 bp), red deer (*Cervus elaphus*; 482) and sambar deer (*Rusa unicolor*; 161 bp). In addition to a no-template control, goat and rabbit DNAs were included as controls to demonstrate PCR specificity

Primer pairs were individually tested using DNA from all six species of mammal. Each primer pair was specific for its respective mammalian species, and the sequences of amplicons were as expected. The multiplexed primers produced amplicons of expected sizes for individual host species (Fig. 1). The amplicons were sequenced and they each matched their respective host on GenBank (Table 2). The original goal was to include all primer pairs into one multiplex PCR. However, this was not possible due to differences in the annealing temperature among some primers. Nonetheless, primer pairs representing all three species of marsupials were combined in the Mmars-PCR and those representing all three species of deer were combined in the Mdeer-PCR.

### Assessment of the multiplex PCR assays

Despite the specificity of primer design, cross-amplification of DNA from other mammals, i.e. rabbits and goats, which consistently occur in water catchments in Victoria, was possible. Thus, the specificity of the primers and the multiplex PCRs were extensively tested. The results (consistent with those presented in Fig. 1) showed that Mmars-PCR was specific for eastern grey kangaroos, swamp wallabies and common wombats, and did not amplify from DNA from deer, goat or rabbit. Conversely, the results showed that the Mdeer-PCR was specific for fallow deer, red deer and sambar deer, and did not amplify *cytb* from marsupial, goat or rabbit DNA.

The analytical sensitivity (lowest level of DNA detection) of each of the two multiplex PCRs was assessed using a ten-fold dilution series (from 1 ng to 0.1 fg) of genomic DNA from muscle. The Mmars-PCR was enabled amplification of *cytb* from a minimum of 0.1 pg of genomic DNA (all species), and the Mdeer-PCR enabled amplification of *cytb* from a minimum of 0.1 pg (red deer) or 1.0 pg (fallow and sambar deer) of genomic DNA.

### Application of the multiplex PCR assays to faecal DNA samples

In total, 669 faecal DNA samples were individually tested in the two multiplex PCR assays. Using these assays, we molecularly authenticated the species of animals for all faecal samples: 419 were from eastern grey kangaroos, 19 from wallabies, 16 from wombats, 163 from sambar deer, 12 from red deer and 10 from fallow deer (Table 3). Using both assays, no amplicons were produced from rabbit or goat DNA. These results showed that some previous inferences of species based on faecal morphology were erroneous. Specifically, 22 faecal samples morphologically inferred to be from kangaroo were from wallaby- or wombat-origin, according to the multiplex PCR results, and one faecal sample inferred morphologically to be from wombat was from a kangaroo. Importantly, none of the 188 faecal samples previously inferred using morphological criteria to be from deer could be linked to a particular species; 3 samples had been inferred to be from kangaroos.

**Table 3:** Summary of the initial morphological identifications of 669 faecal samples collected from Melbourne’s water catchment areas compared with the final PCR results using the two separate multiplex PCRs (Mmars-PCR and Mdeer-PCR), targeting portions of the mitochondrial cytochrome B gene (*cytb*)

Species	Morphological identification	PCR-based authentication
Mmars-PCR		
Unknown marsupial	270	–
Eastern grey kangaroo	168	419
Swamp wallaby	6	19
Mdeer-PCR		
Unknown deer	188^a^	–
Fallow deer	Not possible	10
Red deer	Not possible	12
Sambar deer	Not possible	163
Rabbit	30	No amplicon

^a^Three of faecal samples originally identified morphologically to originate from deer were shown molecularly to originate from kangaroo

### Inferring the geographical origin using genetic data

Interestingly, the three species of deer present within the water catchment areas studied were introduced in the mid-1800s as part of the Acclimatisation Society Initiative [36]. Based on accounts from the literature, sambar deer were thought to have been introduced to Victoria initially in 1862 from Sri Lanka, yet a subsequent introduction from the Philippines was to French Island [36]. The origin(s) of red deer is/are less certain, although they are believed to have been introduced from England [36], which would place them in the Western and Central European clade as opposed to the South Eastern European and Western Asian clades [28]. However, little is known about the origin(s) of fallow deer in Australia, although their populations in Europe were thought to have been heavily anthropologically influenced throughout the Holocene [37]. Thus, it was necessary to first ascertain which clades of deer exist in the catchments prior to designing primers. The *cytb* gene sequence derived from a sambar deer from O’Shannassy, Victoria (accession no. MN746795) was identical to those representing Sri Lankan subclade B (GenBank: MF176994, MF177001 and MF177018; [30]); the *cytb* gene sequence derived from a red deer from Yan Yean, Victoria (accession no. MN746793) was identical to that of the Western and Central European red deer clade (GenBank: KX496937 and MF872247; [38, 39]); *cytb* gene sequence derived from a fallow deer from Cardinia, Victoria (accession no. MN746794) was identical to that from GenBank (JN632629; [27]). All of these sequences were included in an alignment, confirming little sequence variation in the previously designed primers. The results from the *cytb* sequencing of the local deer populations provided the first support that sambar deer populations were originally sourced from Sri Lanka and red deer from England or elsewhere in western Europe [36]. Given the close relatedness of red deer and sambar deer [27], it had been challenging to design primers that differentiated the two species by Mdeer-PCR. Although natural hybrids of the two species have been proposed [40], their existence seems to be unlikely based on experimental evidence [41].

## Conclusions

In the present study, we developed two robust multiplexed PCR assays for the identification and differentiation of marsupial species (common wombats, eastern grey kangaroos and swamp wallabies) and deer species (fallow deer, red deer and sambar deer). These assays are now being used routinely in our laboratory for epidemiological studies and the monitoring of species and genotypes of *Cryptosporidium*, *E. bieneusi* and *Giardia* in these animals in water catchment areas. In the future, we expect that PCR-coupled next generation sequencing (NGS) might be established to expand the number of host species and pathogens to be tested for, depending on time and cost effectiveness. Alternatively, mass spectrometric analysis might also be assessed and implemented, as this approach shows promise for the specific identification of animals, and may also be applicable to the sexing and ageing of animals (cf. [42]).

## Data Availability

Nucleotide sequences reported in this paper are available in the GenBank database under accession numbers MN746793-MN746798.

## Abbreviations

*cytb*: cytochrome *b* gene
EDTA: ethylenediaminetetraacetic acid
Mdeer-PCR: multiplex deer PCR
Mmars-PCR: multiplex marsupial PCR
mt: mitochondrial
NGS: next generation sequencing
PCR: polymerase chain reaction
PCR-RFLP: PCR-based restriction fragment length polymorphism
SDS: sodium dodecyl sulfate

## References

[1] Taberlet P, Waits LP, Luikart G (1999). Noninvasive genetic sampling: look before you leap. Trends Ecol Evol..

[2] Dalén L, Götherström A, Angerbjörn A (2004). Identifying species from pieces of faeces. Conserv Genet..

[3] Waits LP, Paetkau D (2005). Noninvasive genetic sampling tools for wildlife biologists: a review of applications and recommendations for accurate data collection. J Wildl Manag..

[4] Schwartz MK, Luikart G, Waples RS (2007). Genetic monitoring as a promising tool for conservation and management. Trends Ecol Evol..

[5] Nonaka N, Sano T, Inoue T, Armua MT, Fukui D, Katakura K (2009). Multiplex PCR system for identifying the carnivore origins of faeces for an epidemiological study on *Echinococcus multilocularis* in Hokkaido, Japan. Parasitol Res..

[6] Knapp J, Umhang G, Poulle M-L, Millon L (2016). Development of a real-time PCR for a sensitive one-step coprodiagnosis allowing both the identification of carnivore feces and the detection of *Toxocara* spp. and *Echinococcus multilocularis*. Appl Environ Microbiol..

[7] Foran DR, Crooks KR, Minta SC (1997). Species identification from scat: an unambiguous genetic method. Wildl Soc Bull..

[8] Spitzer R, Churski M, Felton A, Heurich M, Kuijper DP, Landman M (2019). Doubting dung: eDNA reveals high rates of misidentification in diverse European ungulate communities. Eur J Wildl Res..

[9] Alvarez G (1994). Morphological variability and identification of deer pellets in central Spain. Folia Zool..

[10] Triggs B (2004). Tracks, scats and other traces: a field guide to Australian mammals.

[11] Bowkett AE, Jones T, Laizzer RL, Plowman AB, Stevens JR (2013). Can molecular data validate morphometric identification of faecal pellets in Tanzanian forest antelope species?. Conserv Genet Resour..

[12] Wadley JJ, Austin JJ, Fordham DA (2013). Rapid species identification of eight sympatric northern Australian macropods from faecal-pellet DNA. Wildl Res..

[13] Costa EBV, de Oliveira ML, Peres PHdF, Grotta-Neto F, Vogliotti A, Piovezan U (2017). Low accuracy of identifying Neotropical deer species by scat morphology. Stud Neotrop Fauna Environ..

[14] Bulinski J, McArthur C (2000). Observer error in counts of macropod scats. Wildl Res..

[15] Alacs E, Alpers D, Paul J, Dillon M, Spencer PB (2003). Identifying the presence of quokkas (*Setonix brachyurus*) and other macropods using cytochrome b analyses from faeces. Wildl Res..

[16] Wadley JJ, Austin JJ, Fordham DA (2014). Genetic inference as a method for modelling occurrence: a viable alternative to visual surveys. Austral Ecol..

[17] Armstrong K, Ball S (2005). DNA barcodes for biosecurity: invasive species identification. Philos Trans R Soc B..

[18] Fajardo V, González I, Rojas M, García T, Martín R (2010). A review of current PCR-based methodologies for the authentication of meats from game animal species. Trends Food Sci Technol..

[19] Bottero MT, Dalmasso A (2011). Animal species identification in food products: evolution of biomolecular methods. Vet J..

[20] Ali ME, Razzak MA, Hamid SBA (2014). Multiplex PCR in species authentication: probability and prospects—a review. Food Anal Methods..

[21] Pun KM, Albrecht C, Castella V, Fumagalli L (2009). Species identification in mammals from mixed biological samples based on mitochondrial DNA control region length polymorphism. Electrophoresis..

[22] Grattarola F, González S, Cosse M (2015). A novel primer set for mammal species identification from feces samples. Conserv Genet Resour..

[23] Gasser RB (2006). Molecular tools–advances, opportunities and prospects. Vet Parasitol..

[24] Nolan MJ, Jex AR, Koehler AV, Haydon SR, Stevens MA, Gasser RB (2013). Molecular-based investigation of *Cryptosporidium* and *Giardia* from animals in water catchments in southeastern Australia. Water Res..

[25] Koehler AV, Haydon SR, Jex AR, Gasser RB (2016). *Cryptosporidium* and *Giardia* taxa in faecal samples from animals in catchments supplying the city of Melbourne with drinking water (2011 to 2015). Parasit Vectors..

[26] Janke A, Magnell O, Wieczorek G, Westerman M, Arnason U (2002). Phylogenetic analysis of 18S rRNA and the mitochondrial genomes of the wombat, *Vombatus ursinus*, and the spiny anteater, *Tachyglossus aculeatu*s: increased support for the Marsupionta hypothesis. J Mol Evol..

[27] Hassanin A, Delsuc F, Ropiquet A, Hammer C, Van Vuuren BJ, Matthee C (2012). Pattern and timing of diversification of Cetartiodactyla (Mammalia, Laurasiatheria), as revealed by a comprehensive analysis of mitochondrial genomes. C R Biol..

[28] Meiri M, Lister AM, Higham TF, Stewart JR, Straus LG, Obermaier H (2013). Late-glacial recolonization and phylogeography of European red deer (*Cervus elaphus* L.). Mol Ecol..

[29] Nilsson MA, Zheng Y, Kumar V, Phillips MJ, Janke A (2017). Speciation generates mosaic genomes in kangaroos. Genome Biol Evol..

[30] Martins RF, Schmidt A, Lenz D, Wilting A, Fickel J (2018). Human-mediated introduction of introgressed deer across Wallace’s line: historical biogeography of *Rusa unicolor* and *R. timorensis*. Ecol Evol..

[31] Edgar RC (2004). MUSCLE: multiple sequence alignment with high accuracy and high throughput. Nucleic Acids Res..

[32] Banks SC, Piggott MP, Hansen BD, Robinson NA, Taylor AC (2002). Wombat coprogenetics: enumerating a common wombat population by microsatellite analysis of faecal DNA. Aust J Zool..

[33] Paplinska JZ, Eldridge MD, Cooper DW, Temple-Smith PD, Renfree MB (2009). Use of genetic methods to establish male-biased dispersal in a cryptic mammal, the swamp wallaby (*Wallabia bicolor*). Aust J Zool..

[34] Eldridge MD, Deakin J, Water P, Graves J (2010). Marsupial population and conservation genetics. Marsupial genetics and genomics.

[35] King WJ, Garant D, Festa-Bianchet M (2015). Mother-offspring distances reflect sex differences in fine-scale genetic structure of eastern grey kangaroos. Ecol Evol..

[36] Bentley A (1998). An introduction to the deer of Australia: with special reference to Victoria.

[37] Baker KH, Gray HWI, Ramovs V, Mertzanidou D, Akın Pekşen Ç, Bilgin CC (2017). Strong population structure in a species manipulated by humans since the Neolithic: the European fallow deer (*Dama dama dama*). Heredity..

[38] Borowski Z, Świsłocka M, Matosiuk M, Mirski P, Krysiuk K, Czajkowska M (2016). Purifying selection, density blocking and unnoticed mitochondrial DNA diversity in the red deer, *Cervus elaphus*. PLoS ONE..

[39] Rey-Iglesia A, Grandal-d’Anglade A, Campos PF, Hansen AJ (2017). Mitochondrial DNA of pre-last glacial maximum red deer from NW Spain suggests a more complex phylogeographical history for the species. Ecol Evol..

[40] Powerscourt V (1884). On the acclimatization of the Japanese deer at Powerscourt. Proc Zool Soc Lond..

[41] Muir P, Semladi G, Asher G, Broad T, Tate M, Barry T (1997). Sambar deer (*Cervus unicolor*) × red deer (*C. elaphus*) interspecies hybrids. J Hered..

[42] Davidson NB, Koch NI, Sarsby J, Jones E, Hurst JL, Beynon RJ (2019). Rapid identification of species, sex and maturity by mass spectrometric analysis of animal faeces. BMC Biol..

